# Time is of the essence for ParaHox homeobox gene clustering

**DOI:** 10.1186/1741-7007-11-72

**Published:** 2013-06-26

**Authors:** Myles Garstang, David EK Ferrier

**Affiliations:** 1 The Scottish Oceans Institute, University of St Andrews, East Sands, St Andrews, Fife, KY16 8LB, UK

## Abstract

ParaHox genes, and their evolutionary sisters the Hox genes, are integral to patterning the anterior-posterior axis of most animals. Like the Hox genes, ParaHox genes can be clustered and exhibit the phenomenon of colinearity - gene order within the cluster matching gene activation. Two new instances of ParaHox clustering provide the first examples of intact clusters outside chordates, with gene expression lending weight to the argument that temporal colinearity is the key to understanding clustering.

See research articles: http://www.biomedcentral.com/1741-7007/11/68 and http://www.biomedcentral.com/1471-2148/13/129

## Homeobox cluster integrity and colinearity

The ParaHox genes consist of the Gsx, Xlox and Cdx families, involved in the anterior-posterior development of the nervous systems and guts of animals.

The discovery of the ParaHox gene cluster in the basal lineage of chordates, the Cephalochordata or amphioxus, revolutionized our understanding about the origins and evolution of the paradigmatic Hox gene cluster, famed for its role in patterning the anterior-posterior axis in animal embryogenesis [1]. Instead of the Hox cluster evolving in isolation as a single homeobox gene cluster that arose via successive tandem duplications of an ancestral UrHox gene, an ancestral ProtoHox cluster seems more likely, this ProtoHox cluster then duplicating or splitting to give rise to the Hox and the ParaHox clusters. This ProtoHox hypothesis is based upon the three ParaHox genes (Gsx, Xlox and Cdx) not only being another example of a homeobox gene cluster, but the genes also being intermingled with the Hox genes in molecular phylogenetic trees, and the ParaHox cluster also exhibiting the phenomenon of colinearity. We now have ParaHox clusters from an echinoderm, the sea star *Patiria miniata*, as well as the hemichordate *Ptychodera flava*, to compare to those of chordates to further resolve the parameters of homeobox clustering, colinearity and the ancestral functions of the ParaHox genes [2,3].

Colinearity can take various guises. In its original formulation colinearity was recognized as the order of the genes along the cluster matching the order of their expression domains along the anterior-posterior axis during embryogenesis: spatial colinearity. Further forms of colinearity have been recognized, such as temporal colinearity, in which the order of the genes along the cluster now corresponds to the order in which the expression of each gene is initiated. As taxon sampling has increased and Hox and ParaHox genes have been isolated from a wider range of species than just the traditional model organisms used in developmental biology, like *Drosophila melanogaster* and the mouse, it has become clear that there is a significant degree of evolutionary flexibility in the organization and function of the Hox and ParaHox genes. ParaHox and Hox genes are not always clustered, and they are not always colinear even when they are clustered. The outstanding questions are why are these genes clustered in some lineages but not others; is this telling us something about the developmental mechanisms in particular species as well as about how the development of that lineage has evolved, and what exactly is the mechanistic basis for these still rather mysterious forms of colinearity?

A vital component in improving our understanding of these homeobox gene clusters and colinearity is to determine the diversity of gene organization and expression across as wide a range of species as possible, in order to discover the pattern that runs through clustering, gene expression and developmental mechanisms. In this vein, two important additions have been made to our battery of taxa in which the organization and expression of the ParaHox genes is known. These are the reports of a ParaHox cluster in the sea star, *P*. *miniata*, from Annunziata *et al*. [2], and the hemichordate *P*. *flava* from Ikuta *et al*. [3] (echinoderms and hemichordates together being known as ambulacrarians). These are the first examples of completely intact ParaHox clusters in animals that are not chordates. Furthermore, the sea star cluster exhibits a significant degree of conservation with the clusters of chordates, both in terms of gene organization and expression, and the hemichordate cluster is particularly notable for the possession of temporal colinearity with only residual spatial colinearity [2,3]. These data help to determine the fundamental, ancestral roles of these genes as well as continuing to tease apart the biology of colinearity.

## ParaHox origins and ancestral roles

**Figure 1:**
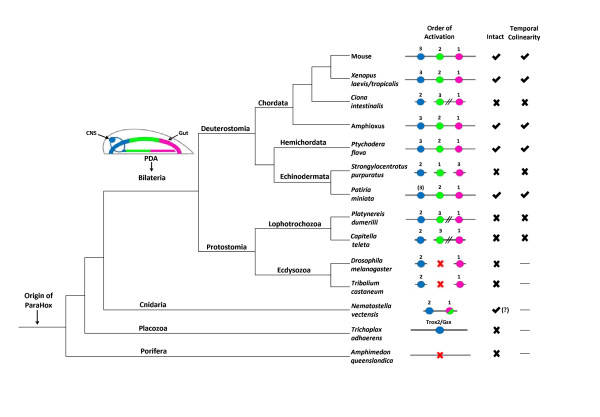
**Animal phylogeny with the correlation between ParaHox cluster integrity and temporal colinearity indicated.** The ParaHox cluster originated before the divergence of the Porifera. The protostome-deuterostome ancestor (PDA) had ParaHox expression in the gut and CNS; with Gsx (blue) anterior, Xlox (green) central and Cdx (pink) posterior (note, this is purely schematic and not intended to illustrate specific morphology or precise expression domain boundaries). Genomic organization of ParaHox genes for each species is shown, with gene linkage represented by a continuous line connecting individual genes. Double diagonals represent genes located on the same chromosome but separated by large distances, and the inclusion of a red ‘X’ indicates loss of one or more ParaHox genes. The *Nematostella* cluster has only 2 ParaHox genes, though it is unresolved whether one of these is a Cdx or Xlox homolog and a third gene has been lost relative to other cnidarians (hence the question mark). The order in which ParaHox genes are activated and expressed has been indicated numerically (*Patiria* Gsx activation in parentheses due to presumed later larval expression). The presence of an intact cluster or temporal colinearity is indicated by a check or cross. A horizontal line indicates that temporal colinearity cannot be resolved due to the absence of one or more ParaHox genes.

The Hox and ParaHox genes originated within the animals, but determining the precise point at which this occurred during animal evolution has been the source of considerable debate. This debate is entwined with controversies about the phylogeny of animals and the resolution of the most basal or earliest branching lineages. Traditionally (as well as in several recent molecular analyses) it is the phylum of sponges, the Porifera, that are recognized as the earliest branching animal lineage. Since no Hox or ParaHox genes have been found in any sponges, it was widely accepted that the ProtoHox state and the subsequent Hox/ParaHox genes did not evolve until later in animal evolution. This has now been thrown into doubt with the discovery of ‘ghost’ Hox and ParaHox loci in the sponge *Amphimedon queenslandica*[4]. These loci are homologous genomic neighborhoods with the Hox and ParaHox loci of higher, bilaterian animals like humans and worms; however, the Hox and ParaHox genes have been lost from these loci in this sponge. This implies that the Hox and ParaHox genes evolved before the origin of the Porifera, in the last common ancestor of all animals (Figure 1). In a similar fashion the single Hox-like gene of the placozoan lineage, *Trox*-*2* in *Trichoplax adhaerens*, has been clearly resolved as a ParaHox gene in a ParaHox locus in this animal, rather than a descendant of the ProtoHox state, as proposed by some authors. *T*. *adhaerens* also has a ghost Hox locus, with conserved synteny to bilaterian Hox loci, but lacking a Hox gene. Moving further up the animal phylogeny into the phylum Cnidaria, species such as *Nematostella vectensis* have both Hox and ParaHox genes present in their Hox and ParaHox genomic neighborhoods. The roles of these homeobox genes in the development of cnidarians is, however, still largely undetermined. There is considerable variability in the expression patterns of the Hox and ParaHox genes in cnidarians, and no clear consensus has been reached as to the ancestral roles of these genes in cnidarians or how these might compare to the functions of the genes in bilaterians.

This is illustrative of the importance and prevalence of gene loss in evolution, as well as evolution of expression, and the dangers of making deductions from a small number of species. Extensive taxon sampling is vital. This is clearly illustrated by the work of Annunziata *et al*. [2], which reveals that the typical model species used to represent the echinoderm condition, the purple sea urchin *Strongylocentrotus purpuratus*, is not so representative after all, at least with regards to the organization and expression profile of the ParaHox genes.

With the expression data from a variety of bilaterian animals, including annelids, molluscs, various vertebrates, amphioxus, urochordates, insects and other echinoderms, the generalities of ParaHox gene expression can be seen to be anterior-posterior domains of expression in both the gut and the central nervous system (CNS), with Gsx being the anterior-most, Xlox the ‘middle’ gene and Cdx the posterior-most expressed ParaHox gene [2]. Consequently, despite the ambiguity about the role of ParaHox genes in cnidarians, we can be certain that the role of the genes in the last common ancestor of the bilaterians (or the protostome-deuterostome ancestor, PDA) was anterior-posterior regionalization of the gut and CNS (Figure 1).

## Is temporal colinearity the key?

As well as these spatial domains of expression and their association with particular tissues and organs there is a notable correlation between the timing of gene activation and the genomic organization of the ParaHox genes. This is now clearly exemplified by the comparison between the echinoderms *S*. *purpuratus* and *P*. *miniata*. In the intact ParaHox cluster of *P*. *miniata* the first gene to be activated is *PmCdx*, followed by *PmLox*, with the final gene *PmGsx* not being expressed in the early larval stages (the bipinnaria) examined by Annunziata *et al*. [2] (note, the low level *PmGsx* expression observed by Annunziata *et al*. is from a maternal contribution and so is not provided from the embryonic ParaHox cluster and is regulated via a different mechanism than whatever produces activation of the embryonic ParaHox genes). This order of expression (Cdx first, Xlox second and Gsx last) matches exactly that of chordates like amphioxus and *Xenopus*[5], and so presumably reflects the order of expression in the last common ancestor of the deuterostomes. This contrasts with the situation in the purple sea urchin, in which the ParaHox cluster has broken apart and *SpLox* is activated first, followed by *SpGsx* and finally *SpCdx* (see Figure five of [6]).

Such a pattern of an intact ParaHox cluster coinciding with temporal colinearity, or similarly a broken ParaHox cluster corresponding to absence of temporal colinearity, is now looking ever more robust. Particularly so since the hemichordate *P*. *flava* now provides us with an example of an intact, ordered cluster that does not have complete spatial colinearity, but does have temporal colinearity [3]. This hemichordate thus highlights the tighter relationship of temporal rather than spatial colinearity with intact, ordered clusters. Intriguingly, this pattern of intact clusters correlating with the presence of temporal colinearity also seems to extend to the Hox gene cluster. This may well reflect the paralogous relationship between the Hox and ParaHox clusters and potentially results from the mechanism that is responsible for temporal colinearity being homologous between the Hox and ParaHox clusters. Obviously more data are required to test this hypothesis and exclude the alternatives: either Hox and ParaHox temporal colinearity arose from distinct mechanisms, or, if there is a common mechanism, then it was co-opted into Hox regulation independently of its co-option into ParaHox regulation. Regardless of which of these alternative evolutionary scenarios is accurate, it seems extremely likely that understanding Hox regulatory mechanisms will inform our understanding of ParaHox mechanisms, and vice versa.

There are already some intriguing similarities, particularly centered on the role of retinoic acid (RA) signaling. Some of the earliest data on regulation of Hox genes revealed a role for RA in sequential temporal activation (for example, in human cell culture [7]), and the direct regulation of Hox genes by RA is well established. Intriguingly, RA regulates all of the ParaHox genes in amphioxus [5]. A link between RA signaling and intact Hox clusters has been proposed [8], which could just as well extend to the ParaHox genes.

Elaborating the precise mechanisms of RA signaling and its role in the regulation of Hox and ParaHox regulation thus has the potential to reveal the basis for temporal colinearity and the evolutionary forces that constrain the integrity of both Hox and ParaHox clusters, possibly entwined with chromatin regulation and progressive movement of cluster regions between inactive and active conditions [9]. We must tread with caution, however, as a distinction must be made between global, pan-cluster regulatory mechanisms as distinct from gene-specific, local mechanisms. And RA may well be involved with both. For example, it is clear that RA regulates Hox1 in *Ciona intestinalis*, whose Hox cluster is largely dispersed, but Cañestro and Postlethwaite [8] propose that this represents a secondarily derived mode of Hox regulation in *Ciona*, which is clearly acting at a gene-specific level. We also need to tease apart the mechanisms producing spatial and temporal control of Hox/ParaHox genes, which can be distinct at least in some contexts in mice [10], and understand these mechanisms in a variety of species. Potentially RA is involved in distinct mechanisms and had an ancient role, since the genes involved in RA signaling are now known to be widespread across the animal kingdom [11]. With this new sea star and hemichordate data the prospect is raised that ambulacrarians could be key systems contributing to this endeavor, with their relatively wide accessibility, abundant embryo and larval material, and a variety of intact versus disorganized and dispersed Hox and ParaHox clusters.
